# Acute Effect of Robotic Therapy (G-EO System™) on the Lower Limb Temperature Distribution of a Patient with Stroke Sequelae

**DOI:** 10.1155/2019/8408492

**Published:** 2019-05-08

**Authors:** Fábio Marcon Alfieri, Caren da Silva Dias, Artur Cesar Aquino dos Santos, Linamara Rizzo Battistella

**Affiliations:** Clinical Research Center, Instituto de Medicina Física e de Reabilitação do Hospital das Clinicas (HCFMUSP), Faculdade de Medicina, Universidade de São Paulo, Rua Domingo de Soto 100, Vila Mariana, 04116-030 São Paulo, SP, Brazil

## Abstract

Robotic therapy has been gaining prominence in poststroke rehabilitation programs. An example of these devices is the G-EO System™, which simulates gait as well as other more complexes standards of gait such as the steps on stairs. However, to the best of our knowledge, there are no studies that apply thermography as a tool to evaluate stroke patients who undertook rehabilitation programs with the aid of robotic devices. The patient IWPS undergoes sequelae of hemorrhagic stroke for 19 months and consequently hemiplegia, had scores of 93 points in the Fugl-Meyer scale, is undertaking a physical rehabilitation program for six months, has no complaints of discomfort due to thermic sensitivity imbalances between the plegic and the contralateral sides, and voluntarily reports that he realizes functionality improvements especially, according to his perception, due to the aid of the robotic therapy in his gait training with the G-EO System™. The thermographic images were captured by an infrared sensor FLIR T650SC. By analyzing the temperature differences between both hemispheres of the body, before, immediately after, and 30 minutes after a robotic therapy for gait training, we observed that the values firstly increased immediately after the training, but after the 30-minute rest an important thermoregulation was achieved.

## 1. Introduction

Along with the conventional treatment, robotic therapies are considered useful to integrate physical rehabilitation programs of patients with sequelae of vascular cerebral accident (stroke) [1]. It is known that the constant activation of the plegic limbs is substantial to the physical rehabilitation process. The robotic therapy aids the movements of the plegic or paretic limbs and may help the functionality recovery, and the robotic devices allow these movements to have greater control as well as the possibility of recovery follow-up [2]. An example of these devices is the G-EO System™ [3], which simulates gait as well as other more complexes standards of gait such as the steps on stairs, even though the scientific literature regarding this device is still not broad enough as to allow conclusions on this system.

Willing to demonstrate possible changes that patients go through during a physical rehabilitation program, a recent study [4] applied thermographic imaging as an assessment tool for evaluating 16 stroke patients. The authors of this study observed that, after the rehabilitation intervention, there was a 0.5°C increase in the temperature of the paretic limb, as well as improvements in the joint functionality; therefore they concluded that thermography can be a useful method for monitoring the effects of a rehabilitation program of patients with stroke. However, to the best of our knowledge, there are no studies that apply thermography as a tool to evaluate stroke patients who undertook rehabilitation programs with the aid of robotic devices.

## 2. Case Presentation

The patient IWPS, a male subject of 25 years of age, white, with body mass index (BMI) of 26.89 Kg/m^2^, undergoes sequelae of hemorrhagic stroke for 19 months and consequently hemiplegia in the right side of the body. He scores 93 points in the Fugl-Meyer scale [5], is undertaking a physical rehabilitation program for six months, has no complaints of discomfort due to thermic sensitivity imbalances between the plegic and the contralateral sides, and voluntarily reports that he realizes functionality improvements especially, according to his perception, due to the aid of the robotic therapy in his gait training with the G-EO System™.

This patient walks with a 1 prong cane and a strap to prevent foot-drop. At the time of the evaluation, he had already held 10 sessions, including simulation of gait training, ascent, and descent of steps. The therapy sessions with G-EO System™ last 20 minutes with initial speed of 0.9km/h and progressive increase of speed up to 1.5km/h. This patient usually achieves an average of 800 steps and 300 steps of stairs simulation, reaching an average of 270m per session. In combination with the robotic therapy, this patient receives the strategic multidisciplinary rehabilitation program, which consists of weekly training sessions of physiotherapy, occupational therapy, physical conditioning, psychology, speech therapy, nutrition, and social and medical services at the Instituto de Medicina Física e de Reabilitação (IMREA). These services assist the patient IWPS on weekly sessions of 50 minutes.

The conventional physiotherapy sessions are composed of stretching and strengthening exercises, mobility and functional training, which consists of active lower limb training at a cycle ergometer, functional electrical stimulation (FES), orthostatism, balance, gait training, and exercises for body perception. Safety training and independence in activities of daily life (ADL) are also included in the program. Robotic and virtual reality therapies are included when prescribed or when the patient is voluntarily included in clinical research protocols [6]. With the objective of evaluating the effect of one training session with the G-EO System™ device over the cutaneous temperature distribution, a case study was conducted with the aid of thermographic imaging.

This evaluation was performed with a protocol of ascending and descending stairs. In this protocol, the patient has his weight supported by a vest and has his feet attached to the system by two platforms, which simulates the gait movements [7]. The cutaneous thermography imaging was captured in the same room of the G-EO System™ at the Instituto de Medicina Física e de Reabilitação (IMREA). All doors, windows, and curtains were closed, so that there was no external light at the patient, and the humidity was 64%. As the conditions for thermography should be standardized, the following steps was observed so as to comply with the specialized literature [8–13].

The patient was told not to take hot shower or bath, not to use ointments or body powder on the skin, and not to perform vigorous physical exercise before the evaluation. The patient should fast for two hours and could not ingest stimulating food, such as caffeine, as well as nasal decongestants, alcohol, and smoke. The patient was requested to wear bathing suit in order to expose the lower limbs and remained in a climatized room at 21.2°C for 15 minutes, so that he could reach a thermic balance with the room before the thermic imaging. The subject could not perform movements like scratching any part of his body. The room temperature was kept at 21.2°C and the windows and curtains were closed to keep out any outside light. The room had cold fluorescent lights. Then, the patient was requested to stand at 4m from the infrared sensor and at 0.4m from the wall [8–13].

The thermographic images were captured by an infrared sensor FLIR T650SC, with resolution of 640 × 480 pixels, with image frequency: 30 Hz, temperature range: - 40°C to 70°C with an accuracy of 1%, spectral range: 7.5 - 14 *µ*m, NETD: <20mK. For the human body, 98% of the body's emissions were considered. Thermal sensitivity of 0.03°C was used with a colorimetric scale (color palette).

The images were in the anterior and posterior incidences of both limbs and were analyzed by the FLIR Tools® software. The mean temperature measurements were determined by anatomical marks of the following regions of interest (ROI): (1) thigh: 5cm above the patella upper border and the inguinal line; (2) leg: 5cm below the patella lower border and 10cm above the malleolus [8].

Other ROIs were also evaluated, such as the calcaneus, the surface of the fifth, and third and first toes, as well as the medial and lateral regions of the feet. For this, the camera was placed at 1.5m, as described by Gatt et al. [14]. The data collection was conducted before, immediately after, and 30 minutes after the robotic session with the G-EO System™. The session in which this case study was conducted lasted 20 minutes and the patient walked 782 steps, 286 of which were on simulated stairs, reaching 241.8 meters.

The temperature data has shown that, among the ROI, only the calcaneus had completely symmetric measurements, given both hemispheres must be symmetric with difference close to 0°C when measured in healthy individuals [15]. Older classifications reported that a difference of 1°C between both sides was considered normal [12].

However, with the technological advances of these devices, a recent study considers evident symmetry when both hemispheres have a difference of temperature of as much as 0.4±0.3°C [16], and another recent study has shown that, among healthy subjects, the largest difference between both sides was 0.49°C, and that the mean difference among 12 body spots was 0.17°C [13]. Nonetheless, in this case report the investigation regards a patient with stroke, a condition that bears thermoregulation issues.

**Figure 1:**
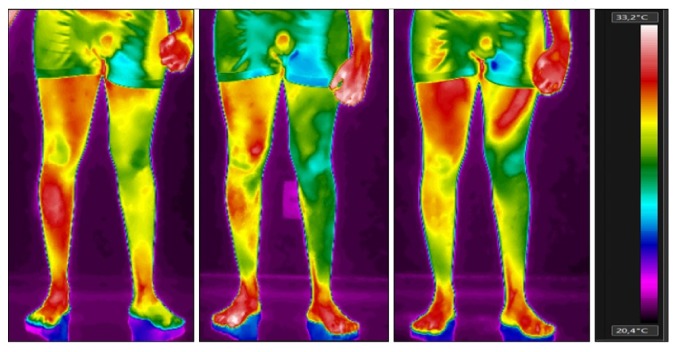
Thermographic image of the anterior regions of thigh and lower leg at baseline, immediately after the robotic training, and 30 minutes after the end of the robotic training (from left to right).

**Figure 2:**
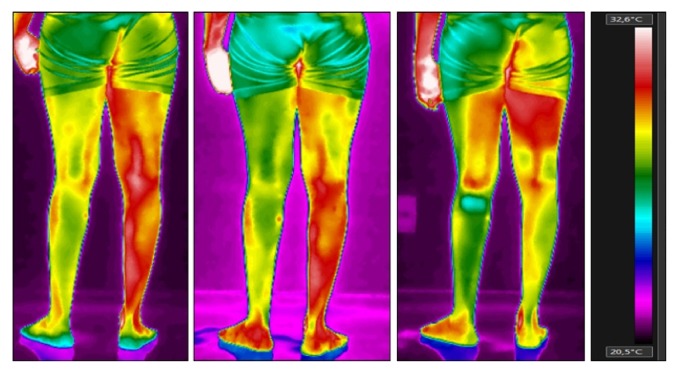
Thermographic image of the posterior regions of thigh and lower leg at baseline, immediately after the robotic training, and 30 minutes after the end of the robotic training (from left to right).

**Figure 3:**
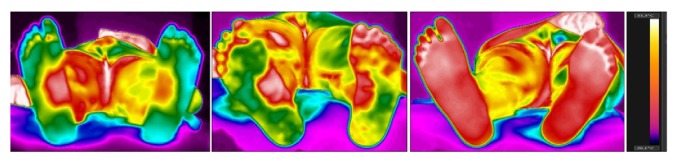
Thermographic image of the plantar cutaneous region at baseline, immediately after the robotic training, and 30 minutes after the end of the robotic training (from left to right).

Table 1 shows the temperature of thigh and lower leg in anterior and posterior views and the measurement values of the evaluated regions. These images can be seen in Figures 1 and 2.  Figure 3 shows the thermographic image of the plantar cutaneous region.

By analyzing the temperature differences at baseline between the plegic and the contralateral side, we observed that only three spots had less than 1°C of difference, all of which were in the feet.

Regarding the thigh and the lower leg, both had more than 1°C of difference, and the lowest temperature was found in the plegic side. Immediately after the intervention, there was an increase in the difference of temperature between both sides of the thigh in the anterior view, most probably due to an indirect increase of cutaneous blood flow caused by the constant muscle contraction. However, after 30 minutes of rest, we observed that the temperature difference was reduced to less than 50% in both sites, the shin and the lower leg, whereas the values found on the thigh revealed that the temperature symmetry could be considered normal, as long as the difference did not exceed 0.3°C. By analyzing the posterior view of the thigh, the asymmetry remained the same, as the temperature difference was approximately 1°C colder in the plegic side. Concerning the lower leg, the posterior and the anterior view have evidenced a decrease in the temperature difference after the robotic training, especially seen in the anterior view, as, after the 30-minute rest, there was a difference of 0.6°C between both the plegic and the contralateral sides.

As for the ROI of the feet, we observed that, before the intervention, the calcaneus had homogenous temperature when both sides were compared, whereas the difference in all the other regions exceeded 0.5°C. Immediately after the intervention, we observed that some specific places, such as hallux, third and fifth toes, and the calcaneus, had their temperature difference increased, as the plegic side experienced an increase of temperature. Nevertheless, after the 30-minute rest, we observed a tendency toward temperature balance between the plegic and the contralateral sides, as the widest difference found was 0.5°C in the third toe and the medial and the lateral regions of the feet, whereas all the other values found (hallux, calcaneus, and fifth toe) could be considered normal, as their difference did not exceed 0.3°C.

A hypothesis explaining this tendency toward temperature normalization between both sides of the plantar region may be the greater weight load on the feet during the gait training by the robotic device, which may suggest that this training allowed an increase in the peripheral blood flow and, therefore, a more efficient thermoregulation causing the temperature to be balanced.

Also, by analyzing the mean temperature difference of the evaluated areas of both sides, we observe that there were 1.23°C before and 1.51°C right after the intervention, whereas the measurements after the 30-minute rest resulted in 0.5°C of difference between both sides; i.e., after the rest period, we observed a tendency toward a normalized distribution of temperature between the plegic and the contralateral sides.

## 3. Discussion

These findings of a single case of one training session and a single follow-up 30 minutes after the intervention do not allow the generalization of a possible long-lasting thermoregulation between the body hemispheres. Nonetheless, these observations raise the question as to whether this robotic device is a useful tool for improving the weight load distribution during the gait training and what could be proven by the recovery of the blood flow balance between both sides. Regarding the use of G-EO System™, only one Pubmed indexed publication is found in the literature. In 2010, Hesse et al. [7] tested the G-EO System™ on 6 subacute stroke patients for 5 weeks and observed that, even though this system is an interesting option for gait recovery training after stroke, their study could not demonstrate the efficacy of this device on the gait of these patients.

Our case report had the objective of verifying the acute effect of the robotic device over the lower limb temperature of the patient, whose thermic imbalances found in the clinical evaluations of thermography are not observed by the patient himself, which confirms previous findings that patients with stroke not always perceive ongoing temperature imbalances [17].

By analyzing the temperature differences between both hemispheres of the body, before, immediately after, and 30 minutes after a robotic therapy for gait training, we observed that the values firstly increased immediately after the training, but after the 30-minute rest an important thermoregulation was achieved, as the temperatures of both sides were as symmetric as the values found in healthy individuals, evidencing the acute effects of this physical exercise. Therefore, we believe that thermography is useful for the immediate evaluation of the alterations caused by the physical exercise stimuli, which is one of the main sources of heat for the human body [12]. In the patient of this case report, the robotic assisted physical exercise not only increased the cutaneous temperature but also caused the differences between the plegic and the contralateral sides to be reduced. How these differences behave in the medium and the long term could not be the objective of this study; however, a prospective clinical trial may raise relevant information regarding the effects of rehabilitation programs with robotic devices on the thermoregulation of patients with stroke sequelae.

## Figures and Tables

**Table 1:** Table 1. Cutaneous temperature distribution of thigh, lower leg, and foot of both plegic and contralateral sides at baseline, immediately after a single session of robotic therapy with G-EO System™, and after a 30-minute rest.

		Baseline	Difference between healthy and plegic sides	Immediate follow up	Difference between healthy and plegic sides	30 minute follow up	Difference between healthy and plegic sides
Anterior view	Healthy thigh	30.0	1.1	28.1	2.0	30.6	0.3
	Plegic thigh	28.9		26.1		30.3	
	Healthy lower leg	30.8	1.8	27.6	1.5	29.0	0.6
	Plegic lower leg	29.0		26.1		28.4	

Posterior View	Healthy thigh	29.3	1.1	28.8	1.0	30.1	1.0
	Plegic thigh	28.2		27.8		29.1	
	Healthy lower leg	30.3	2.3	30.4	2.1	28.8	1.3
	Plegic lower leg	28.0		28.3		27.5	

Feet	Healthy hallux	26.4	1.2	30.2	-1.2	31.5	-0.3
	Plegic hallux	25.2		31.4		31.8	
	3rd healthy toe	24.6	-0.5	28.3	-2.9	31.1	-0.5
	3rd plegic toe	25.1		31.2		31.6	
	5th healthy toe	24.1	-0.8	28.6	-2.1	31.4	0.0
	5th plegic toe	24.9		30.7		31.4	
	Healthy medial	30.8	1.7	31.1	0.5	31.4	0.5
	Plegic medial	29.1		30.6		30.9	
	Healthy lateral	27.4	1.8	26.5	-0.8	31.8	0.5
	Plegic lateral	25.6		27.3		31.3	
	Healthy calcaneus	25.1	0.0	27.0	-1.5	31.0	0.1
	Plegic calcaneus	25.1		28.5		30.9	
